# Breast cancer induced nociceptor aberrant growth and collateral sensory axonal branching

**DOI:** 10.18632/oncotarget.20609

**Published:** 2017-09-01

**Authors:** Matt Austin, Laura Elliott, Niovi Nicolaou, Anna Grabowska, Richard P. Hulse

**Affiliations:** ^1^ Cancer Biology, School of Cancer and Stem Sciences, School of Medicine, University of Nottingham, Nottingham, United Kingdom

**Keywords:** VEGF-A, cancer, breast, MDA MB231, pain

## Abstract

The tumour and neuron interaction has a significant impact upon disease progression and the patients quality of life. In breast cancer patients, it is known that there is an interaction between the tumour microenvironment and the sensory neurons to influence the progression of cancer as well as pain, though these mechanisms still need to be clearly defined. Here it is demonstrated that in a rodent orthotopic model of breast cancer (MDA MB 231) there was an increase in nerve fibre innervation into the tumour microenvironment (protein gene product 9.5), which were calcitonin gene related peptide positive C fibre nociceptors. In contrast, there was a reduction in myelinated nerve fibres (NF200). A sensory neuronal cell line was cultured in response to conditioned media from MDA MB231 and MCF7 as well as vascular endothelial growth factor-A (VEGF-A). All these experimental conditions induced sensory neuronal growth, with increased formation of collateral axonal branches. Furthermore, it was demonstrated that MDA MB231 and VEGF-A induced sensory neuronal sensitisation in response to capsaicin a TRPV1 agonist. MDA MB231 induced neuronal growth was suppressed by VEGFR2 inhibition (ZM323881 and neutralising antibody DC101), in addition both MDA MB231 and VEGF-A induced neurite growth was attenuated by the inhibition of ARP2/3 complex through co-treatment with CK666. This demonstrates that breast cancer can interact with the sensory nervous system to drive neuritogenesis through a VEGF-A/VEGFR2/ARP2/3 mediated pathway.

## INTRODUCTION

Cancer remains one of the main causes of mortality worldwide though survival rates have greatly improved during the last couple of decades, with many instances of cancer survival now above 90%. It is important to appreciate that the burden of the disease lies not only in the mortality rates, but also in those living with this disease. The tumour microenvironment and the tumour-stroma interaction is well-studied to allow for the identification of new cancer treatments; however, one aspect the tumour-neuronal interaction, is often overlooked. The process of cancer growth and metastasis is multi-factorial, with the stroma consisting of a heterogenous population of cell types due to inflammation, angiogenesis or nerve infiltration [1]. Neural invasion of cancer is common (breast [2] and pancreatic [3]), implicating neurons in cancer. Neural invasion of tumour sites is deemed to be an indicator of a poor prognosis, with increased PGP9.5 peripheral nerve fibre innervation associated with aggressive grade III breast cancer [4] and metastasis [5].

Primary nerve fibres play a role in a multitude of physiological functions such as by regulating vascular function (e.g. vasodilation) through the release of pro-angiogenic factors (e.g. calcitonin gene related peptide; CGRP) [6]. Communication between neurons and tumour sites utilise neuropeptides (e.g. nerve growth factor (NGF) [2, 7]) to support those key pathological events (angiogenesis [4] and metastasis [8]) that are key for cancer progression. This is highlighted by tumour induced expansion of sensory nerve fibre innervation, such as in bone metastasis [9], or conversely perineural invasion of migrating cancer cells along nerve fibres utilising the peripheral nervous system to act as a bridge to infiltrate into the central nervous system [10]. Furthermore, one of the key symptoms indicating tumour-nerve interactions is pain, which is especially important for consideration of quality of life as 85% of breast cancer patients survive beyond 10 years [11]. Post-surgical pain is found in up to 53% of women whom have had treatment for breast cancer [12], and for those patients whom their disease has metastasised to bone, pain is also common [13]. Cancer-induced hyperalgesia and breakthrough pain has been strongly associated with the pathological reorganisation of sensory nerves in response to the cancer (breast, pancreatic) environment [9, 14–16]. Aberrant nerve fibre sprouting has been linked to pain in a number of diseases such as arthritis [17] and breast cancer [9], where it is deemed to an indicator of disease progression [4].

Understanding the communication pathways that occur between tumour cells and neurons will allows us to identify key signalling events that cause neuritogenesis [15, 18] of sensory nerve fibres in cancer. We have identified previously that vascular-endothelial growth factor-A (VEGF-A) interacts with sensory neurons [19] and is known to be upregulated in breast cancer [20]. The hypothesis of this study is that breast cancer utilises the innate adaptability of the sensory neuron to respond to an environmental cue to induce sensory neuronal growth and activation. This study highlights that metastatic breast cancer induces sensory neuronal growth via a VEGF-A/VEGF receptor 2-ARP2/3 mediated pathway.

## RESULTS

### Hyperinnervation of nerve fibres in a breast cancer rodent model

**Figure 1:**
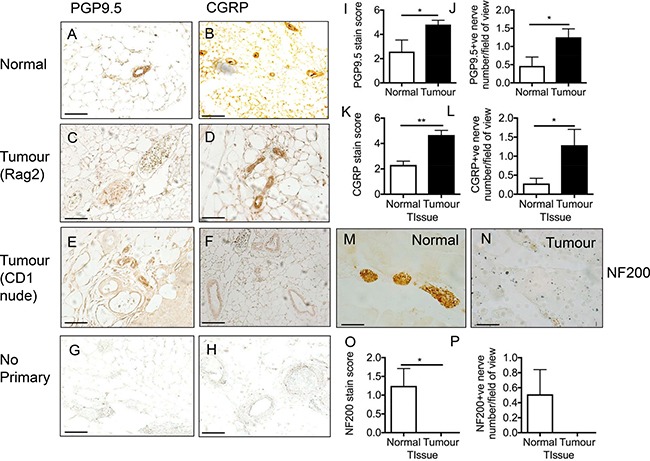
Breast cancer induced sensory nociceptor aberrant growth (**A**) Pan-nerve fibre (PGP 9.5) and (**B**) nociceptor (CGRP) immunoreactivity was present in the mammary tissue of an adult female mouse indicative of nerve fibre innervation. In rodent orthotopic models (representative images of (**C**–**D**) Rag2; [E–F] CD1) of breast (MDA MB231) cancer there was an increase in (**C**, **E**, **I**, **J**) nerve fibre innervation (PGP9.5). (**D**, **F**, **K**, **L**) There was an increase in CGRP positive nociceptor nerve fibre innervation in the tumour. Representative images of no primary controls for (**G**) PGP9.5 and (**H**) CGRP. Myelinated nerve fibre innervation was present in the (**M**) normal mouse though this was decreased in the (**N**–**P**) tumour (CD1). Arrows = nerve fibres, arrowheads = blood vessel. Scale bar = 50 μm.

It has been previously documented that nerve fibres are found at high densities in breast cancer [4]. Rodent models of bone cancer pain derived from breast [9] and prostate [22], have identified hyperinnervation (aberrant sensory nerve fibre growth) of CGRP and NF200 positive sensory nerve fibres in tumourigenic tissue. Furthermore, nerve fibres are present in the mammary gland and have also been demonstrated in the primary breast tumour site [4]. In this study, a breast cancer rodent model of orthotopically injected MDA MB231 cells was used to determine nerve fibre density in breast tumours. Normal mammary gland was compared to those from tumour-bearing mice (Rag2 and CD1 nude). The xenografts were stained with the pan-nerve fibre marker protein gene product 9.5 (PGP9.5; Figure 1A, 1C, 1E) and nociceptor marker calcitonin gene related product (CGRP; Figure 1B, 1D, 1F). Representative no primary controls are presented for PGP9.5 (Figure 1G) and CGRP (Figure 1H). Image analysis showed that in the tumours there was increased immunoreactivity for PGP9.5 (Figure 1I, 1J; nerve number/field of view; Normal = 0.46 ± 0.25; Tumour = 1.25 ± 0.23) and CGRP (Figure 1K, 1L; nerve number/field of view; Normal = 0.29 ± 0.13; Tumour = 1.29 ± 0.41) compared to non-tumour bearing mice. In contrast, tumour-bearing tissue samples stained for NF200 (myelinated nerve marker; Figure 1M, 1N; nerve number; nerve number/field of view; Normal = 0.51 ± 0.33; Tumour = 0 ± 0.00) showed no significant differences with non-tumour bearing tissue (Figure 1O, 1P).

### Breast cancer cell line-conditioned media stimulates sensory neuron growth

**Figure 2:**
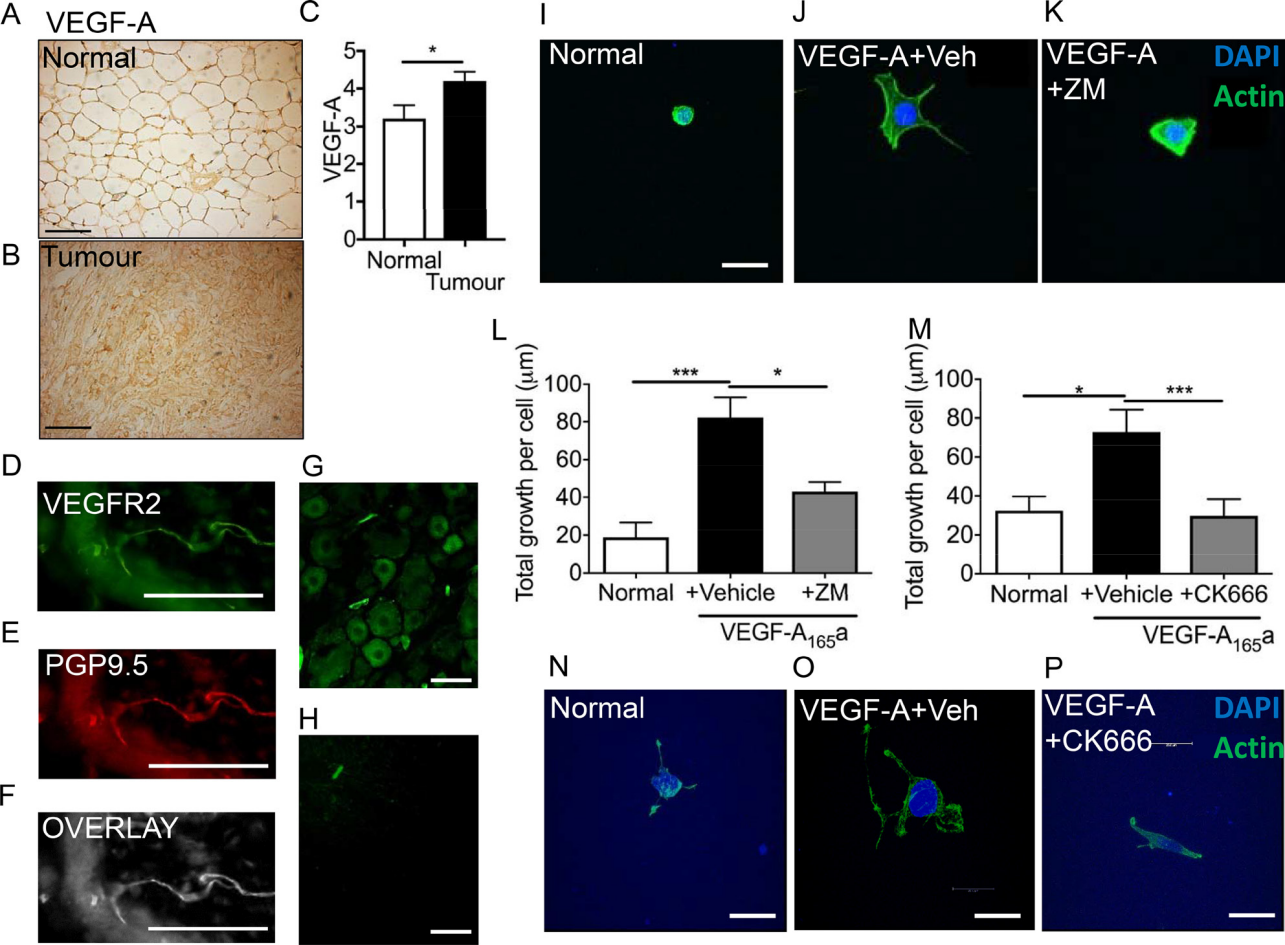
VEGF-A induced sensory neurogenesis mediated by VEGFR2-ARP2/3 (**A**) VEGF-A immunoreactivity in normal mouse mammary fat pad and in an (**B**) MDA MB231 orthotopic model of breast cancer (CD1 mice). (**C**) VEGF-A is upregulated in the breast tumour tissue. (*p < 0.05, Unpaired *T* test). (**D**) VEGFR2 is expressed in (**E**) peripheral nerve terminals (PGP9.5; (**F**) Overlay) in the plantar skin and in (**G**) L5 DRG sensory neurons (scale bar = 50 μm). (**H**) No primary control of L5 DRG sensory neurons. (**I**) Sensory neurons were treated with either (**J**) VEGF-A_165_a + vehicle or (**K**) VEGF-A + ZM323881 and total neuronal growth was measured. (**L**) VEGF-A_165_a led to a significant increase in neuronal growth versus untreated cells. ZM323881 treatment attenuated VEGF-A_165_a induced sensory neuron growth. (**M**) Furthermore, VEGF-A_165_a induced sensory neuron growth was inhibited following CK666 treatment to inhibit ARP2/3 function. Representative images of (**N**) sensory neurons in response to either (**O**) VEGF-A_165_a + Vehicle and (**P**) VEGF-A+CK666. *P < 0.05, ***P < 0.001 Kruskal Wallis with Dunns multiple comparison. Scale bar = 20 μm.

Vascular endothelial growth–A (VEGF-A) expression is known to be upregulated in breast cancer [23, 24] and in the breast cancer cell lines [25]. Using an orthotopic breast cancer model (Figure 2A normal mammary tissue, Figure 2B orthotopic breast cancer tissue) demonstrated an increase in VEGF-A increase in the tumour tissue (Figure 2C; Normal = 3.21 ± 0.35 ID; Tumour 4.20 ± 0.25 ID; ID = integrated density). VEGFR2 is expressed in sensory nerve terminals (Figure 2D) and is colocalised with a pan neuronal marker, PGP9.5 (Figure 2E; Overlay Figure 2F). In addition, it is expressed in L5 sensory DRG neurons (Figure 2G; no primary control Figure 2H) [26, 27]. Sensory neurons were treated with VEGF-A_165_a ± VEGFR2 inhibitor; ZM323881 (Figure 2I–2K; representative images). VEGF-A_165_a treatment induced a significant increase in neurite growth in sensory neurons versus sensory neurons cultured in normal media (Figure 2L; normal media = 18.88 ± 7.82 μm; VEGF-A_165_a+vehicle = 82.14 ± 10.83 μm). VEGFR2 activity was inhibited using ZM323881 which led to suppression of VEGF-A_165_a induced sensory neuron growth (Figure 2L; VEGF-A_165_a+ZM323881 = 43.05 ± 5.01 μm). Furthermore, VEGF-A_165_a induced increasee in total sensory neuron growth when compared to vehicle was significantly reduced by CK666 treatment (Figure 2M; normal media = 32.46 + 7.23 μm, VEGF-A+Vehicle = 72.84 ± 11.38 μm; VEGF-A+CK666 = 29.69 ± 8.57 μm; Representative images Figure 2N; Normal media, Figure 2O; VEGF-A_165_a+Vehicle, Figure 2P; VEGF-A165a+ CK666). Ck666 treatment did not induce cell death (data not shown).

**Figure 3:**
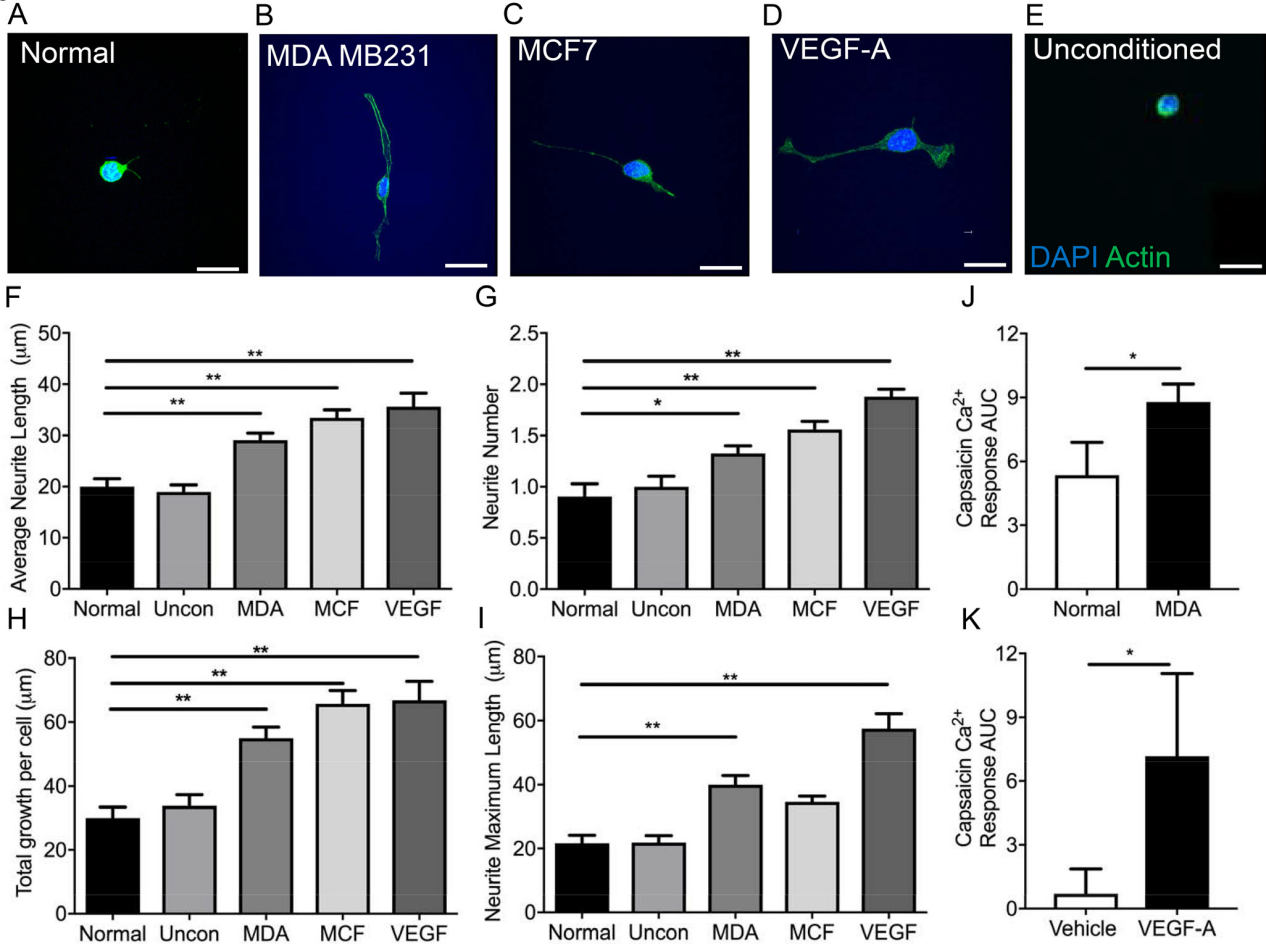
MDA MB231 and MCF7 breast cancer cell lines induce sensory neuronal growth A sensory neuronal cell line (**A**) 50B11 was exposed to conditioned media from (**B**) MDA MB231 and (**C**) MCF7 breast cancer cell lines as well as (**D**) VEGF-A and (**E**) unconditioned media. Scale bar = 20 μm. Conditioned media from MDA MB231 and MCF7 as well as VEGF-A_165_a led to an increase in the (**F**) average neurite length, (**G**) increased neurite number and (**H**) total growth per cell. (**I**) MDA MB231 conditioned media and VEGF-A_165_a led to increased maximum neurite length. (**J**) MDA MB231 conditioned media treated DRG sensory neurons had an increased capsaicin responses when compared to normal media treated DRG sensory neurons. Furthermore, (**K**) VEGF-A_165_a increased capsaicin induced sensory DRG neuronal responses versus vehicle treated DRG neurons (*n* = 8 per experimental group). *P < 0.05, **P < 0.01, ***P < 0.001 Unpaired *T* test or Kruskal Wallis with Dunns multiple comparison. Scale bar = 20 μm.

The sensory neuronal cell line, 50B11, was cultured in the presence of (Figure 3A) normal media, conditioned media taken from (Figure 3B) MDA MB 231, (Figure 3C) MCF-7 or (Figure 3D) VEGF-A_165_a as well as (Figure 3E) unconditioned cancer cell line media for 24 hrs to determine neurite growth. MCF-7 and MDA MB 231 media led to increased average neurite length (Figure 3F; Normal = 19.99 ± 1.53 μm; Uncon = 18.94 ± 1.37 μm; MDA MB231 = 29.08 ± 1.35 μm, MCF = 33.43 ± 1.55 μm; VEGF-A = 35.57 ± 2.696 μm), total neurite number (Figure 3G), total growth per cell (Figure 3H) and maximum neurite length (Figure 3I) when compared to those cells cultured in normal media and unconditioned media. VEGF-A_165_a has previously been shown to induce neurite growth [19], and acts as a positive example in these studies (Figure 3A–3I). Furthermore, MDA MB 231 conditioned media led to sensitisation of DRG sensory neuron responses to capsaicin, a TRPV1 agonist when compared to normal media (Figure 3J; Normal = 5.35 ± 1.55 Ca2^+^ AUC; MDA MB 231 = 8.79 ± 0.84 Ca2^+^ AUC). In addition, VEGF-A_165_a also led to increased TRPV1 responses in DRG sensory neurons when compared to vehicle (Figure 3K; Normal = 0.69 ± 1.17 Ca2^+^ AUC vs VEGF-A = 7.16 ± 3.89 Ca2^+^ AUC) as previously reported [27].

**Figure 4:**
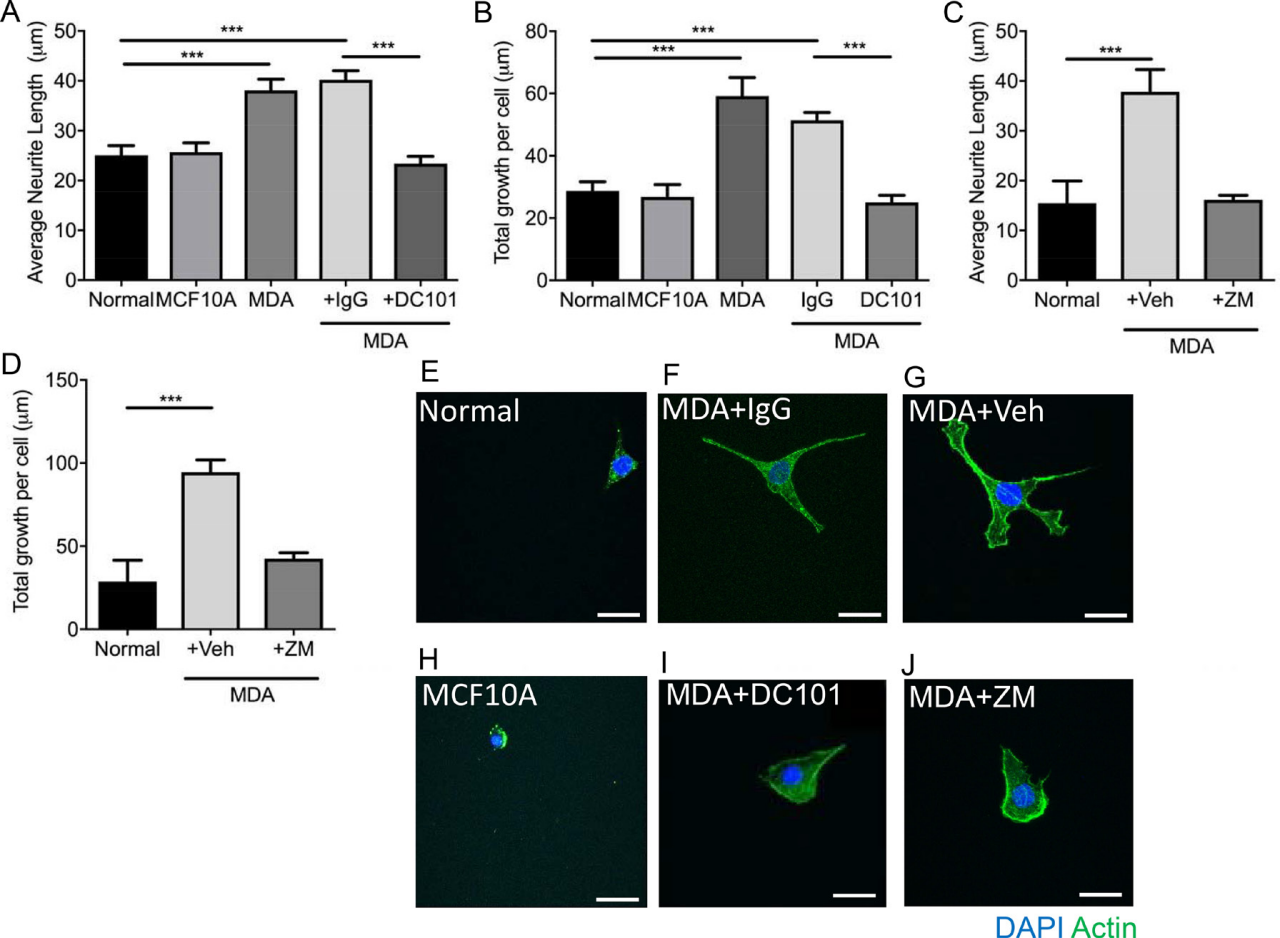
MDA MB231 breast cancer cell line induces sensory neuronal growth via VEGFR2 activation MDA MB231 conditioned media induced an increase in (**A**) average neurite length and (**B**) total neurite growth of sensory neurons when compared to normal media and conditioned media from MCF710A. (A and B) MDA MB231 conditioned media induced sensory neuroritogenesis was inhibited with VEGFR2 neutralising antibody DC101 when compared to rat IgG. Furthermore, inhibition of VEGFR2 using ZM323881 led to inhibition of the MDA MB231 conditioned media induced increase in sensory neuron (**C**) average neurite length and (**D**) total neuron growth. Representative images of sensory neurons in (**E**) normal media conditions, (**F**) MDA MB231 conditioned media + IgG, (**G**) MDA MB231 conditioned media + Vehicle, (**H**) MCF10A conditioned media, (**I**) MDA MB231 conditioned media + DC101 and (**J**) MDA MB231 conditioned media + ZM323881. ***P < 0.001 Unpaired *T* test or Kruskal Wallis with Dunns multiple comparison. Scale bar = 20 μm.

Inhibition of VEGFR2 with DC 101 (rat monoclonal antibody), led to the inhibition of MDA MB231 conditioned media induced neuritogenesis demonstrated by reduced individual neurite length (Figure 4A; Normal = 25.04 ± 1.94 μm; MCF10A = 25.67 ± 1.89 μm; MDA MB231 = 38.1 ± 2.23 μm; MDA MB231+IgG = 40.19 ± 1.82 μm; MDA MB231+DC101 = 23.39 ± 1.43 μm) and total neurite growth (Figure 4B; Normal = 28.71 ± 2.95 μm; MCF10A = 26.79 ± 3.92 μm; MDA MB231 = 59.21 ± 5.92 μm; MDA MB231+IgG = 47.03 ± 2.72 μm; MDA MB231+DC101 = 25.04 ± 2.84 μm). In addition, MCF10A, a normal epithelial breast cell line, conditioned media did not induce neuritogenesis of the sensory neurons (Figure 4A, 4B). Additionally, the VEGFR2 tyrosine kinase inhibitor ZM323881 was used to inhibit MDA MB231 conditioned media induced sensory neuron growth; average neurite length (Figure 4C; Normal = 15.46 ± 4.46 μm; MDA MB231+Vehicle = 37.81 ± 4.47 μm; MDA MB231+ZM323881 = 16.13 ± 0.90 μm) and total sensory neuron growth (Figure 4D) was reduced with ZM323881 treatment (Representative examples of sensory neurons cultured in Figure 4E Normal; Figure 4F MDA MB231+IgG; Figure 4G MDA MB231+`vehicle; Figure 4H MCF10A; Figure 4I MDA MB231+DC101; Figure 4J MDA MB231+ZM323881).

### ARP2/3-mediated MDA-MB231-induced sensory neuritogenesis

**Figure 5:**
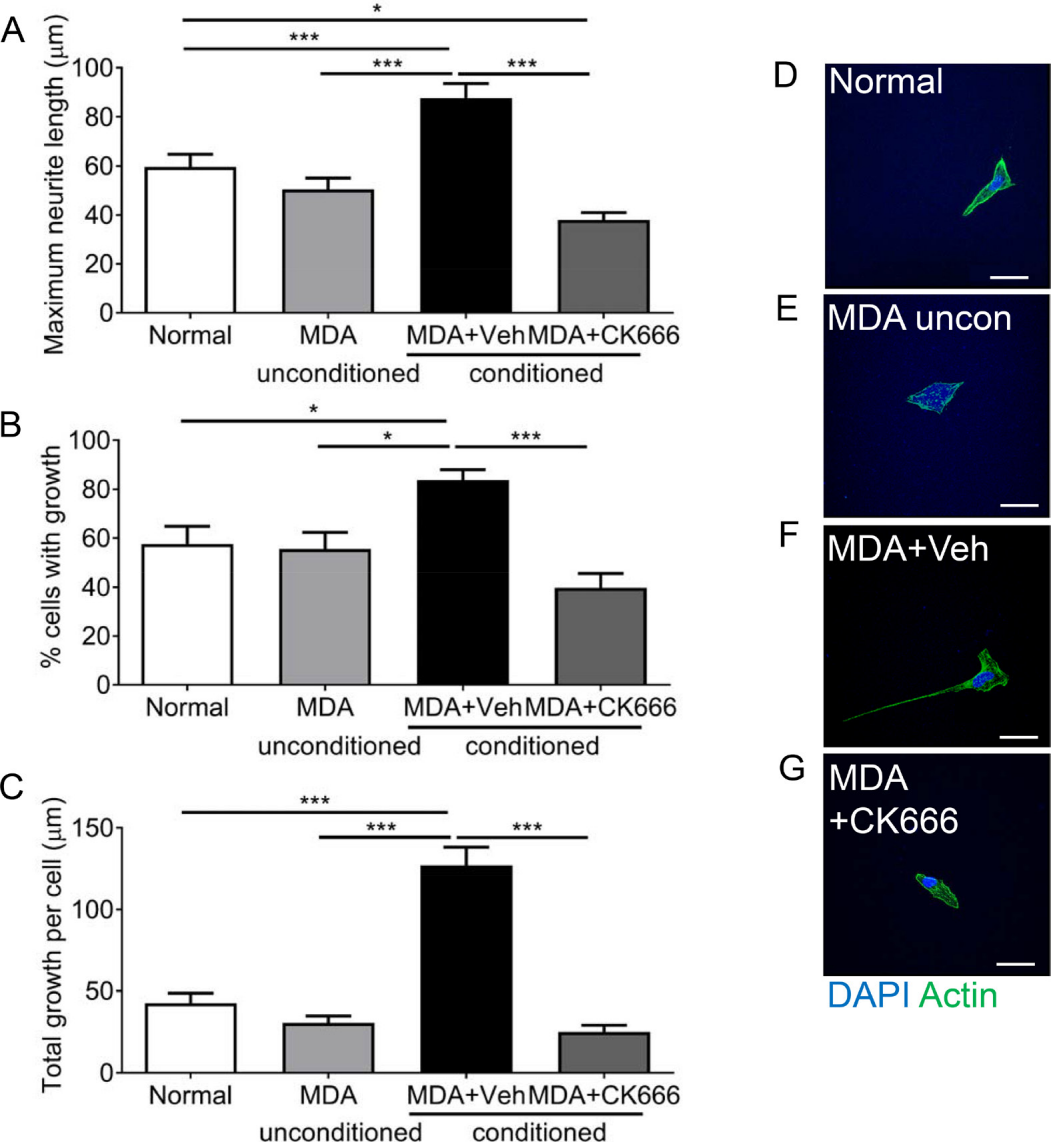
ARP 2/3 regulation controls MDA MB231 induced sensory neuronal growth MDA MB231 induced increase in (**A**) maximum neurite length, (**B**) percentage of neurons with neurites and (**C**) total growth per neuron were attenuated by ARP2/3 inhibitor CK666. Representative images sensory neurons in response to experimental conditions; (**D**) normal media, (**E**) MDA MB231 unconditioned media, (**F**) MDA MB231 conditioned media + Vehicle, (**G**) MDA MB231 conditioned media + CK666. *P < 0.05, **P < 0.01, ***P < 0.001 Kruskal Wallis with Dunns multiple comparison. Scale bar = 20 μm.

MDA MB 231-conditioned media induces neuritogenesis (Figure 3), however evidence supports that ARP2/3 is key to sensory neuron filopodia formation. MDA MB 231 conditioned media led to increased neurite growth of sensory neurons, demonstrated by increased maximum neurite length (Figure 5A), percentage of cells with neurite growth (Figure 5B) and total neurite growth per cell (Figure 5C) when compared to normal media and unconditioned media. CK666 has previously been shown to inhibit neurite formation [28, 29]. Application of 100 μM CK666, ARP2/3 inhibitor, led to a significant reduction in neurite length (Figure 5A; MDA MB231+Veh = 87.65 ± 5.93 μm; MDA MB 231+CK666 = 37.96 ± 2.99 μm), percentage of cells with growth (Figure 5B; MDA MB231+Veh = 83.7 ± 4.26%; MDA MB 231+CK666 = 39.68 ± 5.9%), percentage cells with multiple neurites and total growth per cell (Figure 5C; MDA MB231+Veh = 127.00 ± 11.21 μm vs MDA MB231+CK666 = 25.18 ± 3.74 μm). Representative images of (Figure 5D Normal) sensory neurons in response to (Figure 5E) MDA MB231 unconditioned media, (Figure 5F) MDA MB231 conditioned media+Vehicle and MDA MB231 conditioned media +CK666 (Figure 5G).

### MDA MB231 induced collateral sensory neurite branch formation

**Figure 6:**
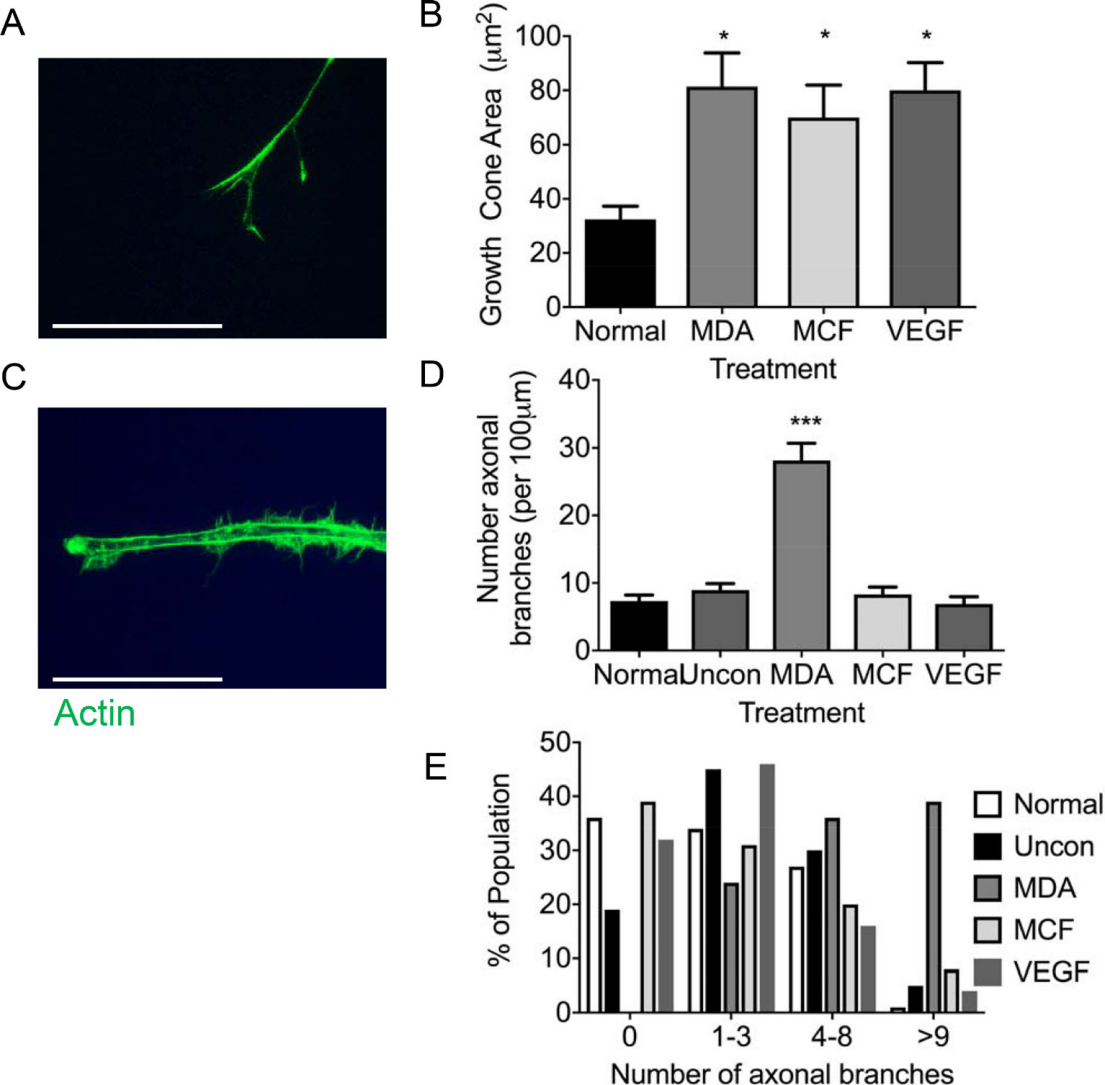
MDA MB231 cancer cells drive sensory neuronal growth cone expansion and collateral branch formation (**A**) Sensory DRG neurons treated with conditioned media from MDA MB231 and MCF7 as well as VEGF-A demonstrated increased growth cone area (Scale bar = 10 μm). (**B**) In addition, the number of collateral branches formed was increased in sensory neurons exposed to conditioned media from MDA MB231 cells when compared to normal media, unconditioned media, MCF7 and VEGF-A. (**C**) This was further demonstrated by MDA MB231 conditioned media inducing a greater percentage of sensory neurons to have greater numbers neurite branches (Scale bar = 20 μm). Representative images of sensory neuron (**D**) growth cone and (**E**) collateral branches. *P < 0.05, ***P < 0.001 Kruskal Wallis with Dunns multiple comparison.

Sensory neuronal filopodia formation via actin and microtubule formation leads to axonal branch formation. Representative example of neurite filopodia F actin rich (phailloidin stained) (Figure 6A). MDA MB 231 conditioned media led to an expansion of neurite growth cone area in relation to normal media (Figure 6B; Normal = 32.43 ± 4.88 μm^2^; MDA MB 231 = 81.36 ± 12.49 μm^2^), with MCF-7 and VEGF-A_165_a also resulting in an increase in growth cone area versus normal media (Figure 6B; MCF = 69.88 ± 12.01 μm^2^; VEGF-A = 79.99 ± 10.25 μm^2^). Collateral branching of the neurite (Figure 6C) was increased (normalised collateral branch number/100 μm neurite length) with MDA MB 231 conditioned media compared to normal and unconditioned media (Figure 6D; Normal = 7.38 ± 0.86 collateral branch number; MDA MB 231 = 28.15 ± 2.55 collateral branch number), while MDA MB231 unconditioned media 8.95 + 0.96 collateral branch number), MCF7 conditioned media (8.32 + 1.11 collateral branch number) and VEGF-A_165_a (6.91 + 1.07 collateral branch number) did not stimulate collateral branch formation (Figure 6D). Furthermore, this is demonstrated by increased number of neurites expressing greater than 9 collateral offshoot branches in the MDA MB 231 conditioned media group compared to other experimental groups (Figure 6E).

### MDA MB 231 induced collateral branch formation is ARP2/3 mediated

**Figure 7:**
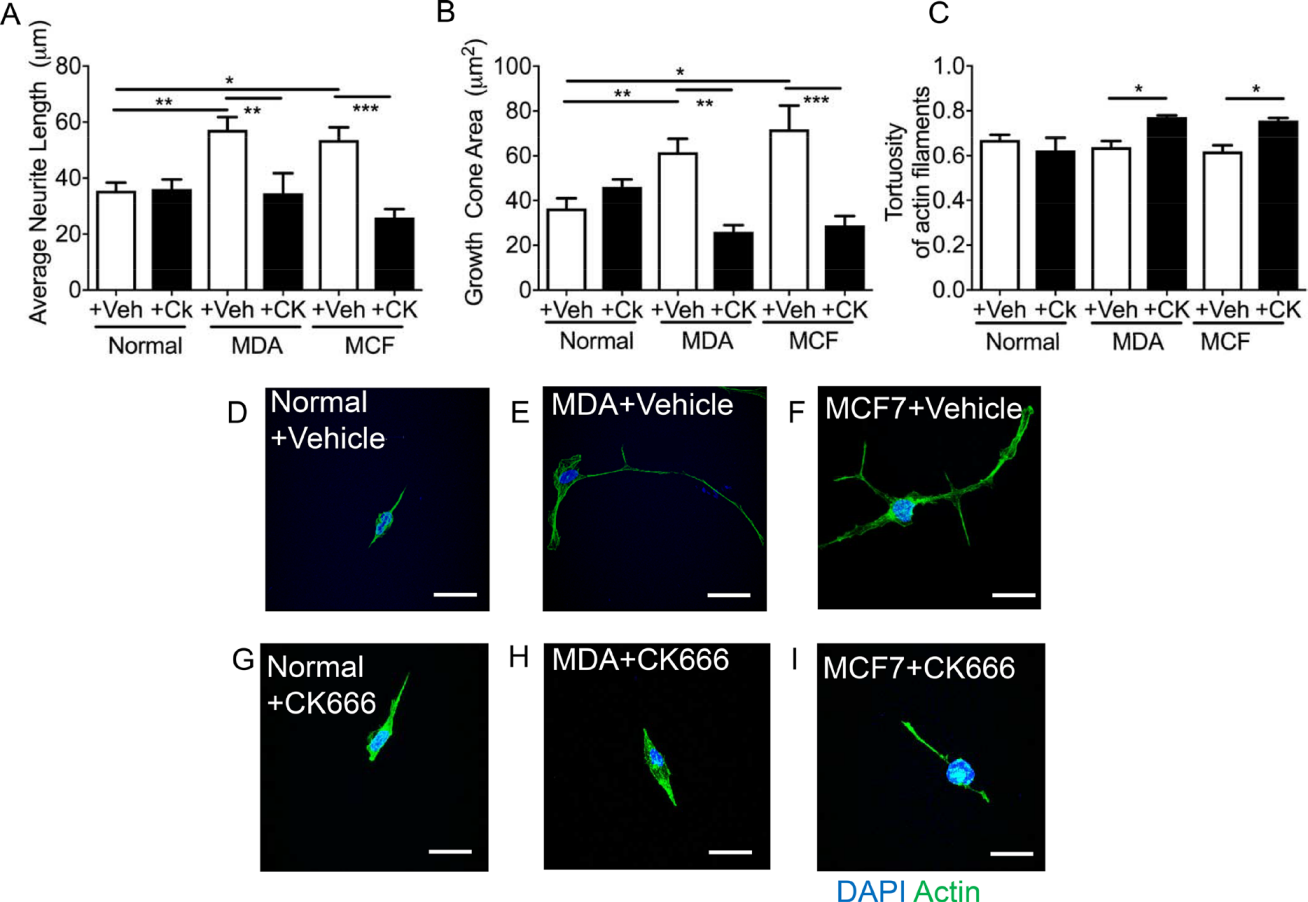
CK666 inhibited sensory neuritogenesis induced by both MDA-MB-231 and MCF7 MDA MB231 and MCF7 induced sensory neuron growth; (**A**) average neurite length and (**B**) growth cone area versus normal media. This was inhibited by CK666 administration. Furthermore, (**C**) the tortuosity of the actin filaments was increased in CK666 sensory neurons indicative of a reduction in actin filament formation and straightness of such fibres Representative images of the sensory neuronal cell line 50B11 under varying experimental conditions; (**D**) normal media + vehicle, (**E**) MDA MB231 conditioned media + vehicle, (**F**) MCF7 conditioned media + vehicle, (**G**) normal media + CK666, (**H**) MDA MB231 conditioned media + CK666, (**I**) MCF7 conditioned media + CK666. *P < 0.05, ***P < 0.001 Kruskal Wallis with Dunns multiple comparison. Scale bar = 20 μm.

**Figure 8:**
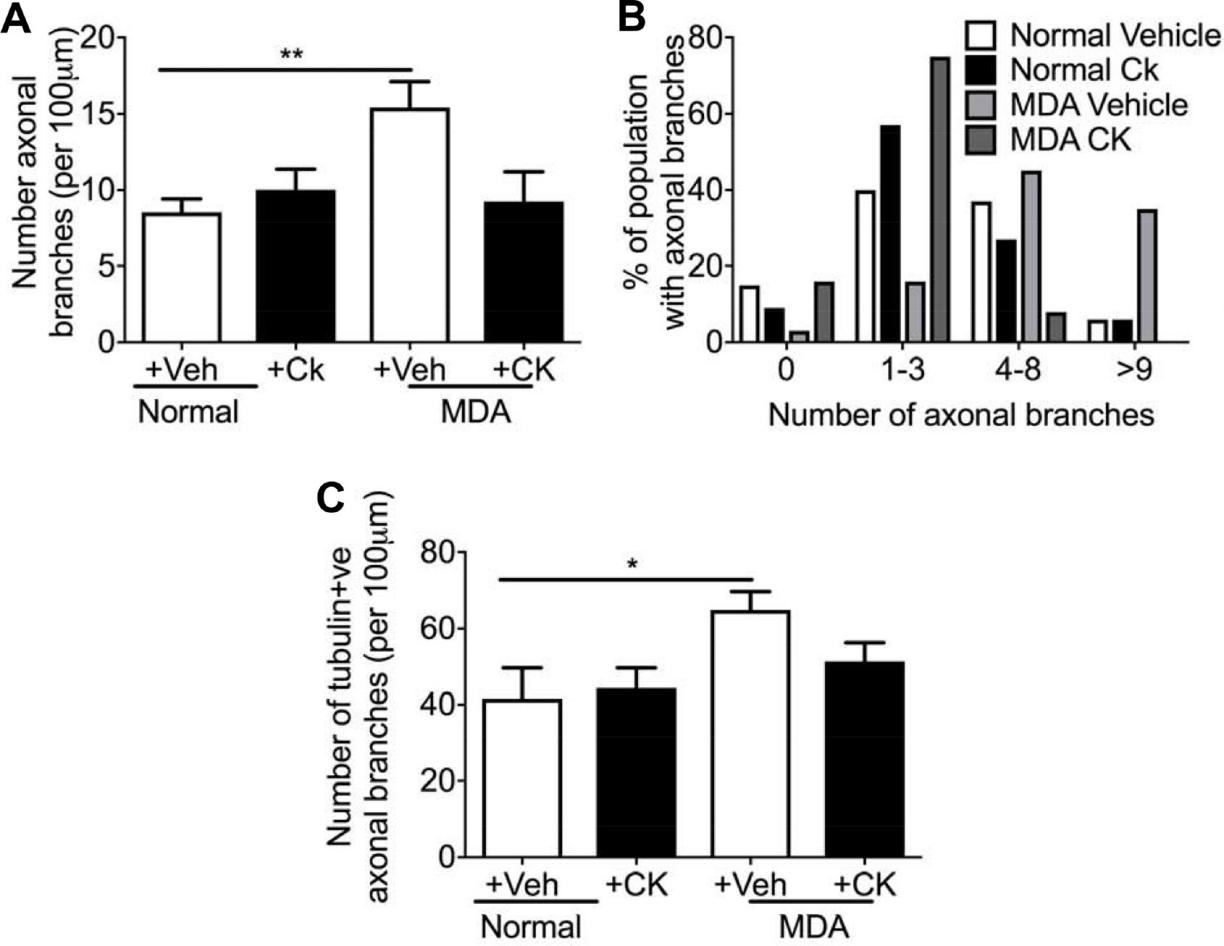
MDA MB231 and MCF7 induced neuritogenesis was inhibited by CK666 administration MDA conditioned media induced (**A**) increased number of collateral branches with (**B**) percentage number of the neuron population had greater than 9 axonal collateral branches. Furthermore, (**C**) treatment of neurons with MDA MB 231 conditioned media increased mature (tubulin positive) axonal branches. ARP 2/3 inhibition with CK666 led to a (A) reduction in total number of collateral branches induced by MDA MB231 conditioned media treatment. In addition, CK66 administration prevented (B) the increase in the % of neurons with greater numbers of collateral branches induced by MDA MB231 conditioned media. (C) MDA MB231 induced mature sensory axonal fibre formation (phalloidin positive/tubulin positive) when compared to normal media treated neurons. CK666 treatment inhibited MDA MB231 induced mature collateral branch formation. *P < 0.05, ***P < 0.001 Kruskal Wallis with Dunns multiple comparison.

**Figure 9:**
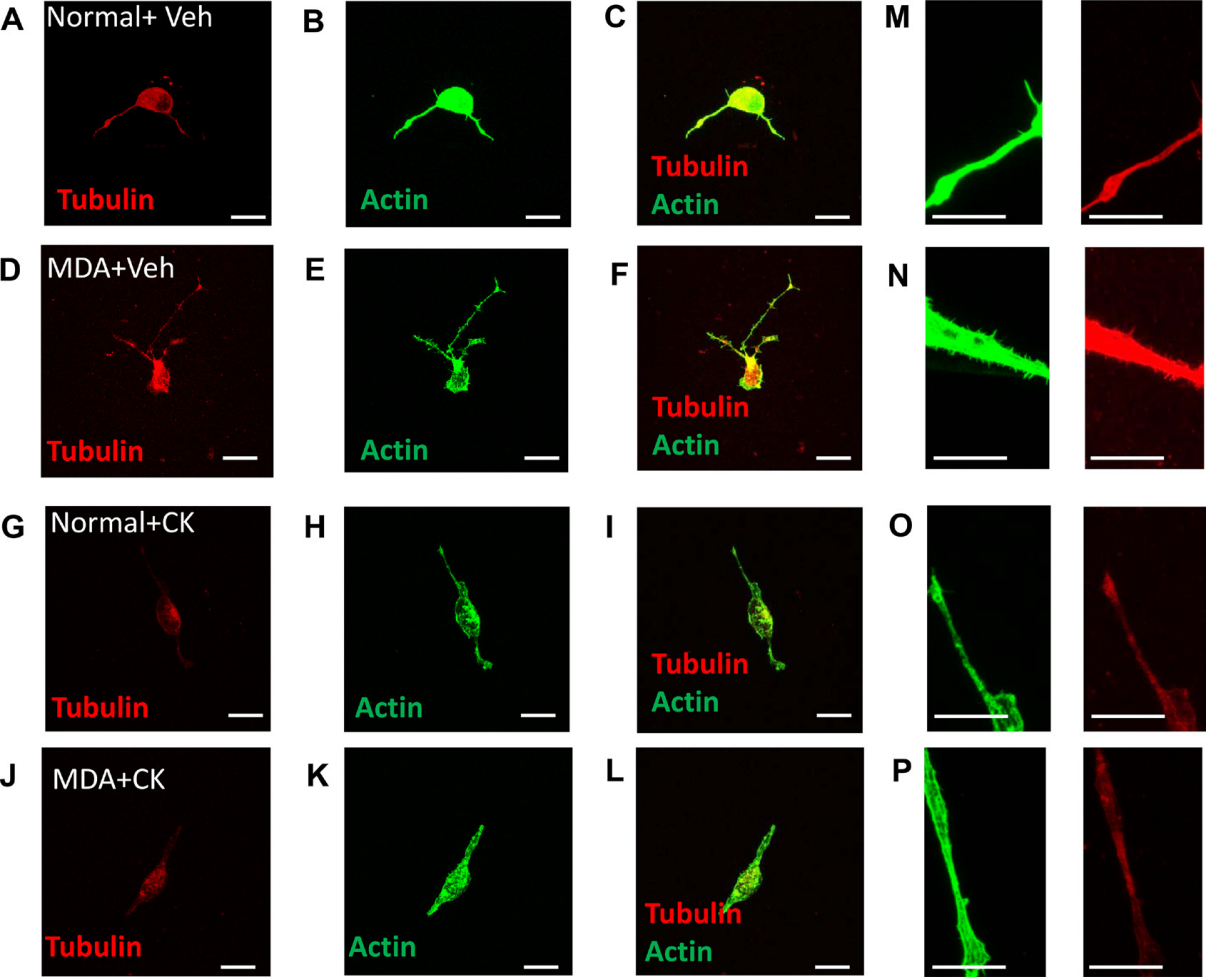
MDA MB231 induced mature sensory axonal neurite formation via ARP2/3 MDA MB231 induced sensory neuronal growth as well as increased neurite formation. Representative images demonstrate that when compared to (**A**–**C**) normal media + vehicle, (**D**–**F**) MDA MB231 conditioned media led to an increase in mature sensory neurite formation (phalloidin positive and tubulin positive). Furthermore, treatment with CK666 did not alter sensory neuronal growth in (**G**–**I**) normal + media but (**J**–**L**) inhibited MDA MB231 induced mature collateral branch formation. High magnification representative images demonstrate that (**N**) MDA MB231 conditioned media induces collateral branch formation when compared to (**M**, **O**) normal media ± CK666 and (**P**) MDA MB231+CK666. Low magnification images = 20 μm (A–L). High magnification images = 10 μm (M–P).

Sensory neuron average neurite length was increased by incubation with MDA MB231 conditioned media versus control (Figure 7A), with co-treatment of MDA MB231 conditioned media with CK666 leading to a significant reduction in average neurite length (Figure 7A; Figure 7B; MDA MB231+Veh = 57.20 ± 4.54 μm; MDA MB231+CK666 = 34.50 ± 7.22 μm; MCF+Veh = 53.68 + 4.45 μm; MCF+CK666 = 25.94 ± 3.06 μm), compared to normal media. There was no change in neurite length in normal treatment treated with CK666 (Figure 7A). MCF-7 induced neurite growth was also inhibited by CK666 treatment (Figure 7A). Both MDA MB231 and MCF-7 induced neurite growth cone area were inhibited by CK666 treatment (Figure 7B; MDA MB231+Veh = 61.66 ± 6.07 μm^2^; MDA MB231+CK666 = 26.08 ± 2.97 μm^2^; MCF+Veh = 71.81 ± 10.63 μm^2^; MCF+CK666 = 28.94 ± 4.19 μm^2^), with normal media being unaffected by CK666 treatment (Figure 7B). Tortuosity of actin filaments were reduced by CK666 treatment in MDA MB 231 and MCF-7 conditioned media treated neurons (Figure 7C; MDA MB 231+Veh = 0.64 ± 0.03 Tortuosity ratio; MDA MB 231+CK666 = 0.77 ± 0.01 Tortuosity ratio; MCF+Veh = 0.62 ± 0.03 Tortuosity ratio; MCF+CK666 = 0.76 ± 0.01 Tortuosity ratio). Representative images of all experimental groups ± CK666 (Figure 7D normal ± vehicle; Figure 7E MDA MB231 ± Vehicle; Figure 7F MCF7 ± Vehicle; Figure 7G Normal ± CK666; Figure 7H MDA MB231 ± CK666; Figure 7I MCF7+CK666). MDA MB231 induced collateral neurite branch formation was decreased by ARP2/3 inhibition through CK666 inhibition (Figure 8A, 8B; Representative images of all experimental groups (Figure 9). Additionally, this was represented by a CK666 inhibition of MDA MB 231 induced increase in the number of collateral branches per neurite (Figure 8A; MDA MB231+Veh = 15.42 ± 1.68 vs MDA MB231+CK666 = 9.25 ± 1.93). There was no difference when comparing vehicle and CK666 treatment in normal media (Figure 8A, 8B). MDA MB231 led to an increase in the number of collateral branches that were positive for tubulin (Figures 8C, 9A–9NA) therefore these were classified as mature axonal collateral branches as previously termed [28]. CK666 inhibited MDA MB 231 induced tubulin positive collateral branch formation (Figures 8C, 9G–9PG; MDA MB231+Veh = 64.97 ± 4.67% vs MDA MB231+CK666 = 51.39 ± 4.85%).

## DISCUSSION

The role of the neuro-tumour microenvironment is still unclear. It is appreciated that the nervous system interacts with the tumour, leading to advancement of breast cancer [2, 4]. Furthermore, evidence now demonstrates that this interaction may have further implications in relation to tumour support and metastasis [1, 5, 30]. Investigating the interaction between cancer and the sensory neuron is key to providing a greater depth of understanding in cancer biology. In this study, the interaction between breast cancer and the sensory neurons is explored to determine the mechanisms that underlie breast cancer induced neuritogenesis.

Understanding the neuro-cancer interaction is crucial as it has been implicated in disease progression with increases in nerve innervation present in the primary breast tumour site [4]. Current literature demonstrates a significant neuro-cancer interaction whereby in human and rodent studies, there is an expansion of sensory nerve innervation patterns in tumourigenic tissue, which is inclusive of breast [5, 9] and pancreatic [31] cancer. This is strongly associated with pain. Here we present that nerve fibres (PGP9.5+ive) innervate breast tumour sites (MDA-MB231), with these determined to be nociceptive sensory neurons (CGRP+ve). This is comparable to previous studies [9, 14, 15]. However, in contrast to the literature [22], myelinated NF200+ve nerve fibres did not innervate the tumour in this breast cancer rodent cancer model, though our study investigates a primary site versus the published work which is a metastatic site.

The increase in PGP9.5-positive staining in the breast tumour supports the notion that cancer induces nerve fibre growth, however the mechanisms by which these interactions occur are still being elucidated. NGF is a key candidate in the pathological growth and aberrant sprouting of neurons in cancer, and has been shown to be responsible for increased pain in cancer [9, 14]. The fact that sensory nerves elongate in response to these tumour derived mediators may have implications for the way in which we understand cancer biology. Rather than simply causing the sprouting of existent nerves around the tumour, these mediators may also cause nerves to grow into the tumour, moving up NGF and VEGF gradients [42] as well becoming hyperactive as demonstrated here through enhancement of sensory neuronal TRPV1 activity. This would induce peripheral nociceptor sensitisation and lead to the development of chronic pain in cancer [32]. In addition, our study highlights not only an increase in free nerve endings and sensory nerve activity but also associations with increased immunoreactivity with blood vessels. Increased nerve activity induces increased vascular permeability via for example CGRP [6, 30] and such activity has been linked to cancer disease progression [30]. Therefore it is plausible that breast cancer growth and migration, could be suppressed following inhibition of neuronal vascular interactions.

Cancer is associated with angiogenesis, immune cell infiltration and in case of bone metastasis skeletal fractures. Majority of work to date concentrates on *in vivo* studies utilising a heterogeneous population of cell types that encompass the tumour stroma [9, 22, 30] highlighting the importance of understanding the nervous system involvement in cancer biology. However these do not take into account specific mechanisms by which these pathologies develop. Such tumour environments can lead to sensory nerve compression and sensory nerve terminal interaction with inflammatory and cancer cell types. These factors are all contributory to neurogenesis with a great body of work highlighting this in cancer pain, however still many questions remain unanswered. Stereotypically in human and rodent studies cancer pain is attributable to activation of the peripheral sensory neuron [32]. A number of inflammatory mediators; that have expression increased in cancer and accompany cancer disease progression, are granulocyte-macrophage colony-stimulating factors (GM-CSF) [15], VEGF-A [27], nerve growth factor (NGF) [33] and glial cell derived neurotrophic factor (GDNF) [34] are known to induce sensory neuron activation and pain; neuropathic and inflammatory pain [15, 27, 35, 36]. Both VEGF-A and NGF sensitise peripheral sensory nerve fibres to a number of sensory stimuli [27, 33] and for example lead to activation of heat sensing channel, transient receptor vanilloid 1 channel TRPV1 [27]. Despite being best known as a pro-angiogenic factor, recent research has shown that VEGF-A is not restricted to its angiogenic capabilities [27]. Previous studies demonstrate that VEGF-A can drive sensory neuronal growth [19, 37] and is neuroprotective [38, 39]. Studies presented here demonstrate that those tumour derived mediators directly drive sensory neuronal function (growth and sensory neuron TRPV1 activation) in a VEGF-A dependent manner. In addition, MDA MB231 and MCF7 cancer cells induce sensory neurite growth. Furthermore, inhibition of VEGFR2 through either ZM323881 or DC101 treatment inhibited MDA MB231 induced neurite growth highlighting that VEGF-A/VEGFR2 signalling is an important regulator of breast cancer induced sensory neuritogenesis. Therefore such a VEGF-A enriched tumour environment would propose to induce neuronally mediated CGRP nerve fibre branching, hyperactivity and induce angiogenesis for tumour support.

However, there are a multitude of mediators and mechanisms that regulate sensory neuronal function, many of which have not been explored in relation to cancer-neuron interactions. It is important to note that many have investigated the molecular components of sensory neurite growth in relation to neuroregeneration and synaptogenesis in the nervous system, primarily targeting NGF induced TrkA activation which in turns leads to neurite growth [40]. NGF drives neurite growth through expansion of the axonal growth cone as well as directionality by targeting NGF rich areas [29, 40]. In addition to increases in axonal length, NGF drives neurite branching along the axonal main trunk creating collateral nerve growth [41]. The formation of such collateral branching is due to the emergence of axonal filopodia from actin rich patches. It has been shown that the ARP2/3 complex provides the framework to initiate such filopodia expansion, with NGF inducing increasing frequency of such sites along the sensory nerve axon [28, 41]. This supports the notion that NGF drives expansion of the sensory nerve fibres in tumours. As well as MDA MB231 and MCF7 induced neurite growth, the growth cone area is also increased with these parameters of sensory neuron growth also associated with VEGF-A treatment. It is highly plausible that additional tumour derived factors are associated with this as NGF secretion is not significantly different between MCF-7 and MDA MB 231 cells [42]. Expression levels of proteins secreted by MDA MB231 such as VEGF-A [43, 44] and insulin-like growth factor binding proteins are higher than in MCF-7 [45] and this is implicated in collateral branch formation. Previous literature has suggested that ARP2/3 is crucial for actin polymerisation, and therefore for sensory neurite elongation and directionality [29]. Therefore in an NGF rich environment, as is produced by many tumours [9], ARP2/3 activation is liable to be widespread with increase frequency in actin rich/ARP2/3 axonal patches [28], ultimately leading to increased neurite growth and branching. CK666 inhibition prevented MDA MB231, MCF7 and VEGF-A neurite growth as well as reducing MDA MB231 and MCF7 induced growth cone expansion highlighting regulation of VEGF-A induced neurogenesis. Additionally, ARP2/3 allows for the formation of filamentous actin, however CK666 treatment led to a reduction in the formation of F actin structures. Surprisingly, MDA MB231 led to increased collateral branching from the sensory nerve axon, whereas VEGF-A and neither MCF7 induced increases in collateral branching. This was reduced by CK666 treatment, with the number of mature branches that had been penetrated by tubulin also reduced. MDA MB231 release greater levels of VEGF-A versus MCF7 [44, 46] therefore it would be expected that MDA MB231 to induce increased neurite formation as it is demonstrated that VEGF-A actions via VEGFR2 induce sensory neuron growth. However, VEGF-A alone did not induce increased collateral branching. This could implicate as mentioned a cocktail of mediators driving sensory neuritogenesis such as NGF, which as mentioned induces collateral neuronal branching, as well as plausibly epidermal growth factor (EGF) or GM-CSF. Sensory DRG neurons express EGF receptor [47] and it has been shown that TGFα induces sensory activation [48] and neuronal growth [49], where as EGF does not [49]. Furthermore, additional growth factors TGFβ inhibits neuronal growth [50] and GM-CSF stimulates neurogenesis and bone cancer pain [15]. These alternative factors such as GM-CSF can differ between these pathologically distinct breat cancer cell lines such as in comparing basal like MDA MB231 and luminal A MCF7 with distinct GM-CSF derived actions demonstrated in MDA MB231 [51]. This differential expression profiles of growth factors provides an explanation for differential induction of collaternal branch formation, and highlights the complex nature of these neuro-cancer interactions which we begin to decipher in this study.

These studies highlight that the tumour environment directly acts upon the sensory neuron to drive alterations in sensory neuronal function through VEGF-A/VEGFR2 induced sensory neuronal activation and aberrant neuronal growth. By identifying these signalling pathways it allows for further understanding of cancer biology, providing crucial evidence in how the sensory neuron-tumourigenic interface controls cancer and pain development.

## MATERIALS AND METHODS

All procedures involving the mice were performed in accordance with the United Kingdom Animals (Scientific Procedures) Act 1986, the UK Home Office and ARRIVE guidelines under project license authority PPL40/3559 using archived tissue. Ethical approval was granted by the University of Nottingham Animal Welfare and Ethical Review Board. CD-1 nude female mice at 6 to 8 weeks of age were obtained from Charles River, UK and Rag2−/− female mice. The animals were provided food and water ad libitum. MDA-MB-231 cells, with viability of > 90%, which had been maintained *in vitro* in RPMI culture medium (Sigma, UK) containing 10% (v/v) heat inactivated foetal bovine serum (Sigma, Poole, UK) & 2 mM L-glutamine (Sigma, UK) at 37°C in 5% CO_2,_ were be re-suspended, for *in vivo* administration, in sterile growth factor reduced matrigel at 2 × 10^6^ cells/100 ul (1 to 2 × 10^7^ cells per ml) and 100 ul of cell suspension was injected into the mammary fatpad for tumour initiation. For primary cell culture calcium experimentation C57Bl6 male mice were used.

### Immunohistochemistry

MDA-MB-231 xenografts grown in the mammary fat pad of Rag 2−/− and CD1-nude mice or tissue from uninjected animals as control tissue were fixed in formaldehyde and then dehydrated in sequential concentrations of methanol, followed by immersion in xylene and paraffin wax (BDH, cat 36107 7E). Sections were cut at 10 μm thickness at approximately 30 μm intervals and mounted onto Thermo Scientific Menzel-Gläser Superfrost microscope slides. The slides were then incubated overnight at 40°C.

For immunohistochemistry sections were immersed in xylene for 15 minutes before twice being immersed in 100% methanol. These were washed in distilled water twice, each for five-minute intervals. The slides were then submerged in citrate buffer (pH 6.0) and heated at 98°C for thirty minutes. Sections were then placed under the running water prior to use. Sections were washed three times in PBST (0.2% triton x-100) for five minutes. Subsequently, 3% H_2_O_2_ was applied to each slide for five minutes and then washed with PBS. This step was repeated. Slides were then exposed to PBST for five minutes prior to incubation in blocking solution (5% FBS and 10% BSA in PBST) for one hour at room temperature. Sections were then washed three times in PBS for five minutes. Primary antibodies were prepared in blocking solution and sections were incubated overnight at 4°C. Primary antibodies used were: rabbit anti-VEGF-A (1 in 100 Santa Cruz A20), rabbit anti- calcitonin gene related peptide (CGRP: 1:2000; EMD Millipore) and rabbit anti- protein gene product 9.5 (PGP9.5; 1:250; EMD Millipore). Slides were then washed in PBS three times for five minutes per wash. Sections were incubated with donkey biotinylated anti rabbit IgG (1:500; Jackson) in PBST for 90 minutes at room temperature. ABC reagent was prepared according to the instructions given in the Vectastain ABC kit (Vector Laboratories) and left to stand for 30 minutes. Sections were washed a further three times with PBS for five minutes. Vectorstain was applied to the slides and left for thirty minutes at room temperature. Slides were washed in PBS washes three times. The DAB stain (Vector Laboratories) was prepared as indicated in the given instructions and applied to each slide for two minutes. Each slide was then immediately washed in distilled water for five minutes. Slides were then washed twice in methanol, followed by xylene for 10 minutes. DPX was applied to the slides and coverslips were applied. Slides were imaged using a Leica DMLB microscope at 40× objective and images were collected using Leica QWin V3 software.

### Immunofluorescence

Dorsal root ganglia Lumbar (L) 5 and plantar surface of hindpaws were excised and immersion fixed in 4% paraformaldehyde. This was followed by tissue immersion in 30% sucrose for 24 hours 4^°^C. Tissue was frozen in OCT and stored at −80^°^C until processing. Tissue sections were cut to thickness of 6 μm for dorsal root ganglia and 20 μm for plantar skin. Slides were washed in PBS and was followed by PBST for five minutes prior to incubation in blocking solution (5% FBS and 10% BSA in PBST) for one hour at room temperature. Sections were then washed three times in PBS for five minutes. Primary antibodies were prepared in blocking solution and sections were incubated overnight at 4^°^C. Primary antibodies used were: rabbit anti- vascular endothelial growth factor receptor 2 (VEGFR2: 1:100; Cell Signalling) and mouse anti- protein gene product 9.5 (PGP9.5; 1:10; Abcam). Slides were then washed in PBS three times for five minutes per wash. Sections were incubated with secondary antibody (1:1000; anti-rabbit alexafluor 488 or anti-mouse alexafluor 555) in PBST for 90 minutes at room temperature. Slides were washed in PBS and mounted with vectorshield (Vector Laboratories). Images were acquired using a Leica SPE confocal microscope.

### Neuronal growth assay

A sensory neuronal cell line, 50b11s, was used as previously described [19, 21]. Cells were cultured in Neurobasal Media (ThermoFisher Scientific) supplemented with 10% FBS, 0.5 mM L-Glutamine, 1× B27 and 0.2% glucose. A 24 well plate had cover slips inserted and sterilised with ethanol. 5000 cells were placed in each well and were left for 24 hours prior to incubation in experimental culture condition. In each case 75 μM Forskolin was added as previously described [19]. Experimental groups for conditioned media experiments were as follow:- (a) 400 μl complete neurobasal media, (b) 400 μl unconditioned media, (c) 400 μl MDA conditioned media, (d) 400 μl MCF10A-conditioned media, (e) 400 μl MCF-7-conditioned media, or (f) 400 μl 2.5 nM VEGF in complete neurobasal media. Conditioned media was extracted from cells having been exposed to either MDA MB231 (RPMI supplemented with 10% FBS and 2 mM l-glutamine), MCF-7 (Phenol Red free RPMI supplemented with 10% FBS and 2 mM l-glutamine) or MCF10A (HuMEC serum free medium supplemented with Bovine Pituitary Extract (BPE), HuMEC supplement and 100 ng/μl cholera toxin) cells for 3 days. All wells were made up to 800 μl by adding 400 μl supplemented neurobasal media. Drugs applied were DC101 8.05 μg/ml (versus equivalent rat IgG control) (BioXcell), VEGF-A (2.5 nM R&Dsystems), ZM 323881 (100 nM Tocris) or CK666 (100 μM Sigma-Aldrich). Experimental repeats were a minimum of 4 wells per plate, with a minimum of 3 plates per experiment.

### Immunocytochemistry

Experimental plates were cultured for 24 hours and 4% paraformaldehyde was then applied for 15 minute. Wells were then washed in PBS three times with PBS cells prior to incubation in blocking solution (PBS + 0.2% Tritonx-100, 5% BSA and 10% FBS) for 30 minutes. Phalloidin alexafluor 488 was made up in 0.2% triton in PBS (1 in 500 in block solution; Life Technologies) and added to the slides. For tubulin and actin imaging; cells were incubated in 4% paraformaldehyde (made up in PHEM buffer; 60 mM PIPES, 25 mM HEPES, 10 mM EGTA, 2 mM MgCl_2_, pH6.9) for 15minutes. Phalloidin Alexfluor 488 was added in addition to anti alpha tubulin (mouse; 1 in 500) for 1 hr. Coverslips were washed three times with PBS. For tubulin imaging anti-mouse Alexfluor 555 (1 in 10000, Life Technologies) was added in PBS 0.2% triton X-100). Coverslips were washed three times with PBS. Coverslips were removed and placed on slides with Fluorsave (DAPI containing, Sigma). Slides were imaged using a Leica SPE Confocal Microscope.

### Calcium imaging

Dorsal root ganglia (DRG) sensory neurons were dissected from 4 mice per plate. Each assay contained a minimum of 8 wells per condition with each experiment repeated (minimum of 8 mice per preparation). All DRGS were incubated in F12 media (supplemented with 1ml Penicillin/Streptomycin, 15% BSA and N2). DRGs were added to F12 media and 0.125% collagenase (Sigma) for 2 hrs at 37°C. DRGs were triturated to form a cell suspension, which was then added to the top of a 15% bovine serum albumin solution. This was spun at 1900 rpm for 10 minutes. Supernatant was disposed and pellet was resuspended in supplemented F12 media. 2000 cells per well were added to a 96 well plates that had been treated with poly L lysine and laminin. Cells were left for 48 hrs prior to administration of experimental conditions (100 μl per well): 2.5 nM VEGF-A_165_a and MDA MB231 conditioned media. Fluo4 direct assay (supplier) was used as according to manufacturer's instructions. Fluo4 was made up 10 ml with the supplied Fluo4 direct calcium assay buffer. 5 mM probenecid was added. 60 μl of media was removed following 24 hrs exposure to experimental conditions. 40 ul of fluo4 was then added and left for 1 hr at 37^°^C. The plate was then placed in a Victor Perkin Elmer plate reader. A baseline measurement was recorded from each well prior to drug stimulation. 20 μl of vehicle or 5 μM capsaicin (final working concentration 1 μM) was added to each well. Fluorescence emission was recorded and analysed.

### Statistical analyses section

All data are represented as mean ± SEM and n numbers are presented in the accompanying figure legends. All results were analysed using Graphpad Prizm v7, Microsoft Excel and Image J. At least 5 sections per animal were taken from the normal and tumour bearing samples. The total number of CGRP or PGP9.5 positive nerve fibres were counted per acquired image, and was represented as an average per animal. VEGF-A immunoreactivity was measured using integrated density (Image J plugin). Calcium responses were determined by calculated normalised baseline (F_0_ responses) and post drug capsaicin (F_1_) induced responses from obtained raw data values. Consequently drug responses (F_1_) were determined as a fold change from F_0_ and represented as AUC.

All *in vitro* experimentation was carried out via acquisition of a minimum of 5 images per well. A minimum of 4 wells per experimental replicate was carried out and these studies were repeated. Neurite was determined as a phalloidin stained extension from the cell body, with all measurements taken from this reference point. Neurite parameters (average length, total neurite growth, neurite number and longest neurite) were all determined from images analysed using Image J. Image scale was determined and ImageJ calibrated accordingly. Growth cone area was determined as an outlined area defined in Image J. The number of phalloidin stained collateral branches/offshoots extending from the neurite were counted and normalised to length of neurite to allow for number per 100 μm neurite length. This was due to experimental conditions leading to alterations in neurite length thus the normalisation allowed for direct comparison between groups. The number of phalloidin and tubulin positive neurites were counted and presented as the percentage of phalloidin positive neurites that were also tubulin positive.

## Abbreviations

VEGF-A: vascular endothelial growth factor A
VEGFR2: vascular endothelial growth factor receptor 2
DRG: dorsal root ganglia
PGP9.5: protein gene product 9.5
NF200: neurofilament 200
TRPV1: transient receptor potential vanilloid 1
ARP2/3: actin related proteins 2/3
CGRP: calcitonin gene related peptide
NGF: nerve growth factor
PBST: phosphate buffered saline, triton x-100
BSA: bovine serum albumin
GMCSF: granulocyte macrophage colony stimulating factor
GDNF: glial cell line derived neurotrophic factor
TrkA: tropomyosin receptor kinase A
F actin: filamentous actin
EGF: epidermal growth factor
TGF: transforming growth factor
